# Artificial Intelligence for Cone-Beam Computed Tomography in Endodontics: A PRISMA-Aligned Narrative Synthesis of Evidence From the United States and Europe (2021-2026)

**DOI:** 10.7759/cureus.110786

**Published:** 2026-06-13

**Authors:** Volodymyr Kachmar

**Affiliations:** 1 International Dental Program, Tufts University School of Dental Medicine, Boston, USA

**Keywords:** artificial intelligence, cone-beam computed tomography, deep-learning, endodontics, image segmentation, periapical periodontitis, root canal segmentation, systematic review

## Abstract

Cone-beam computed tomography (CBCT) is the reference three-dimensional imaging modality for endodontic diagnosis and treatment planning, but its interpretation is time-consuming and varies between observers. Artificial intelligence (AI) and convolutional neural networks in particular have been applied to CBCT to automate root canal segmentation, periapical lesion detection, and morphologic classification. Currently, there is no focused synthesis of evidence from the United States and Europe. This review is a Preferred Reporting Items for Systematic Reviews and Meta-Analyses (PRISMA) 2020-aligned narrative synthesis. PubMed/MEDLINE, Scopus, Web of Science, Google Scholar, and IEEE Xplore were searched for primary studies published between January 1, 2021, and April 30, 2026. Studies were included if they used AI with CBCT for endodontic applications and had a first or corresponding author from an institution in the United States or Europe, and quality was appraised against the Checklist for Artificial Intelligence in Medical Imaging (CLAIM) and Quality Assessment of Diagnostic Accuracy Studies tailored to Artificial Intelligence (QUADAS-AI). Of 612 records screened, 17 studies were included, originating mainly from four research groups (the University of Pennsylvania, KU Leuven, the Medical University of Graz, and Stony Brook University), with two further single-study contributions. Tooth, pulp, and canal segmentation was the most mature task (Dice 0.85-0.97), and periapical lesion detection reached early clinical validation (sensitivity 0.80-0.97; specificity 0.84-1.00). No eligible studies from the United States or Europe were found on vertical root fracture detection, AI-based working length determination, American Association of Endodontists case difficulty assessment, or treatment outcome prediction. CBCT-based endodontic AI in these regions is advancing unevenly: segmentation and lesion detection are approaching clinical use, while several clinically important tasks remain unaddressed and external validation across devices is still limited.

## Introduction and background

Apical periodontitis is among the most prevalent chronic inflammatory diseases worldwide, with a pooled individual-level prevalence of approximately 52% in a meta-analysis of 33 studies [1]. Endodontic treatment is the principal intervention for teeth affected by pulpal and periapical disease. Treatment success hinges on three things - accurate diagnosis of pulpal and periapical status, complete identification of root canal anatomy, and recognition of complicating features such as root fractures, resorptive defects, and periapical bone loss. Each of these determinations is inherently three-dimensional, and two-dimensional periapical radiography systematically underestimates lesion extent and misses anatomic variation [2].

Cone-beam computed tomography has therefore become integral to contemporary endodontic practice. Joint position statements from the American Association of Endodontists and the American Academy of Oral and Maxillofacial Radiology (AAE/AAOMR, last updated in 2015) [3] and from the European Society of Endodontology (ESE, 2019 update) [4] now anchor CBCT use in endodontics. Both endorse CBCT as a problem-solving modality - for complex anatomy, suspected vertical root fracture (VRF), persistent or atypical apical pathosis, dental trauma, internal or external resorption, and pre-surgical planning - with imaging restricted to the smallest field of view (FOV) consistent with the clinical question. The European SEDENTEXCT guidelines [5] similarly emphasize justification, optimization, and limited-FOV acquisition.

Despite these advances in image acquisition, CBCT interpretation remains a substantial bottleneck. Apical lesions of less than 4 mm are missed by experienced clinicians at non-trivial rates, interobserver agreement for the periapical index on CBCT (CBCT-PAI) is moderate at best [2,4], and full segmentation of pulp-canal anatomy from a high-resolution volume can require 30-60 minutes per tooth by a trained operator [6-8]. Clinically, undetected anatomy translates directly into morbidity - missed canals are associated with a 4.38-fold increase in the odds of post-treatment apical periodontitis [9], and missed second mesiobuccal (MB2) canals in maxillary molars are a leading cause of root canal treatment failure [10].

Deep learning (DL) has transformed medical image analysis since 2015, when LeCun, Bengio, and Hinton outlined the field's modern foundations [11] and Ronneberger, Fischer, and Brox introduced the U-Net architecture for biomedical segmentation [12]. The self-configuring nnU-Net framework has subsequently become the reference benchmark for 3D medical segmentation [13], and transformer-based models such as Swin-UNETR have begun to challenge CNN dominance even when training data are scarce. Dental AI has followed suit, with regulatory clearance now granted to several products. Multiple dental AI products from companies including Pearl, Overjet, and Diagnocat (Diagnocat Inc., San Francisco, CA, USA) have received US Food and Drug Administration (FDA) 510(k) clearances in recent years, with labeled indications spanning anatomical visualization, segmentation, measurement, and computer-aided detection on intraoral, panoramic, and cone-beam computed tomography imaging [14]. However, these systems are best understood as clinician-assistance tools rather than autonomous endodontic diagnostic systems, and their labeled indications vary substantially across products. The persistent gap between research performance and regulatory clearance remains.

Multiple reviews have surveyed AI in endodontics, including narrative and scoping syntheses by Aminoshariae et al. [15], Umer and Habib [16], Setzer et al. [17], Mohammad-Rahimi et al. [18], Ourang et al. [19], Asgary [20], Khanagar et al. [21], Boreak [22], Fontenele and Jacobs [23], and a diagnostic test accuracy meta-analysis of periapical radiolucency detection by Sadr et al [24]. These contributions are valuable, but they predominantly mix imaging modalities (panoramic, periapical, CBCT), aggregate global literature without consideration of regulatory or population context, and rarely focus on CBCT-specific endodontic tasks at the level of architecture, dataset, and reporting quality.

This review addresses that gap. It systematically synthesizes evidence published between 1 January 2021 and 30 April 2026 on AI applied to CBCT for endodontic segmentation, detection, and classification tasks, restricted to studies with first or corresponding authors at US or European institutions. The geographic restriction reflects two methodological priorities: alignment with the regulatory environments (FDA, Conformité Européenne-Medical Device Regulation (CE-MDR)) most relevant to North American and European readers, and a deliberate audit of whether US and European academic centers have produced a CBCT-specific endodontic AI evidence base proportionate to their visibility in this field. The review's objectives are threefold: to map the current state of CBCT-based endodontic AI in this geography by task; to appraise methodological rigor and reporting against contemporary AI standards (the Checklist for Artificial Intelligence in Medical Imaging (CLAIM), Quality Assessment of Diagnostic Accuracy Studies tailored to Artificial Intelligence (QUADAS-AI), and Transparent Reporting of a Multivariable Prediction Model for Individual Prognosis or Diagnosis-Artificial Intelligence (TRIPOD-AI)); and to identify high-priority research gaps for the next phase of clinical translation.

## Review

Materials and Methods

Reporting Framework

This review was conducted as a Preferred Reporting Items for Systematic Reviews and Meta-Analyses (PRISMA 2020)-aligned narrative synthesis [25]. A formal meta-analysis was not performed because between-study heterogeneity in tasks, ground-truth definitions, dataset sizes, validation strategies, and performance metrics precluded meaningful quantitative pooling. The protocol was developed a priori but was not registered with PROSPERO or comparable registries; this is acknowledged as a methodological limitation below.

Systematic and Narrative Components

The systematic component of this review consisted of a predefined clinical question, database search, eligibility criteria, date range, geographic restriction, two-stage screening, structured data extraction, and PRISMA 2020-style reporting of study flow [25]. The narrative component consisted of qualitative synthesis by task category rather than quantitative meta-analysis. A meta-analysis was not performed because the included studies differed substantially in clinical task, model architecture, ground-truth definition, dataset size, validation strategy, and reported performance metrics.

Eligibility Criteria (PICO-Adapted)

The eligibility framework is summarized below.

Population: patients undergoing CBCT imaging for endodontic indications, or extracted/cadaveric teeth scanned by CBCT for endodontic-relevant analysis.

Intervention: any AI methodology, including classical machine learning, CNNs, recurrent networks, transformers, or hybrid pipelines, applied to the CBCT image as the primary input.

Comparator: expert human interpretation, manual segmentation, micro-computed tomography, surgical or histologic confirmation, or another AI model.

Outcomes: quantitative diagnostic or technical performance metrics including Dice similarity coefficient (DSC), intersection over union (IoU), Hausdorff distance (HD), accuracy, sensitivity, specificity, area under the receiver operating characteristic curve (AUC), positive and negative predictive values (PPV/NPV), and F1 score.

Study designs: original peer-reviewed journal articles or high-quality conference proceedings (Medical Image Computing and Computer-Assisted Intervention (MICCAI), Institute of Electrical and Electronics Engineers (IEEE)).

Studies were excluded if they used non-CBCT modalities (panoramic, periapical, intraoral, or medical CT), addressed non-endodontic tasks (orthodontic, implant-only, or maxillofacial fracture analyses), reported only qualitative outputs, were narrative or systematic reviews, or had first and corresponding authors affiliated exclusively with non-US/non-European institutions. The Republic of Türkiye was treated as borderline and admitted only when an explicit European co-corresponding author was present.

Information Sources and Search Strategy

**Figure 1:**
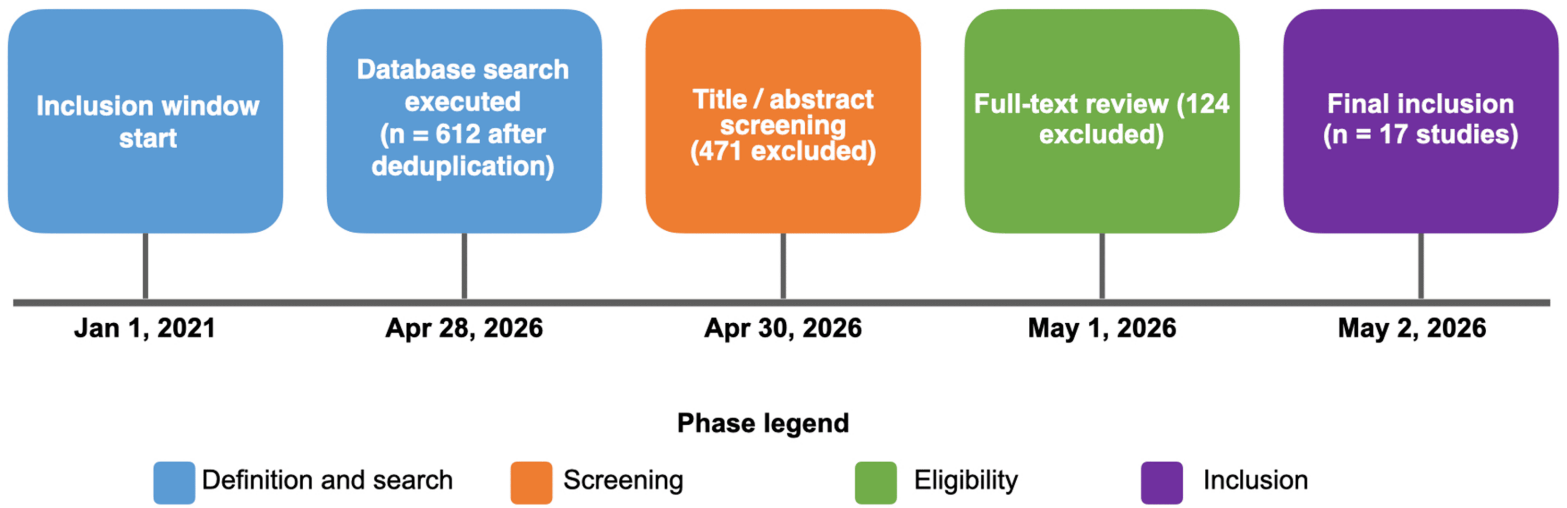
Timeline of search/screening/inclusion Timeline of search, screening, and inclusion process. The figure shows the inclusion window (January 1, 2021 - April 30, 2026), the final database search date (April 28, 2026), title and abstract screening (471 records excluded), full-text review (124 records excluded), and final inclusion of 17 primary studies. Color shading indicates the four PRISMA phases: blue for definition and search, orange for screening, green for eligibility, purple for inclusion. Figure created by the author in Apple Keynote on macOS (Apple Inc., Cupertino, USA). No generative artificial intelligence was used. PRISMA: Preferred Reporting Items for Systematic Reviews and Meta-Analyses

PubMed/MEDLINE, Scopus, Web of Science, Google Scholar, and IEEE Xplore were searched on 28 April 2026. The PubMed strategy combined MeSH and free-text terms: ("Cone-Beam Computed Tomography"[MeSH] OR CBCT) AND ("Deep Learning"[MeSH] OR "Artificial Intelligence"[MeSH] OR "Neural Networks, Computer"[MeSH] OR "convolutional neural network" OR transformer) AND (endodontic* OR "root canal" OR pulp OR "periapical lesion" OR "apical periodontitis" OR "vertical root fracture" OR "C-shaped" OR MB2 OR Vertucci OR "working length"). For Scopus, Web of Science, IEEE Xplore, and Google Scholar, equivalent free-text queries were adapted from this strategy, since MeSH headings are unique to PubMed and MEDLINE; the same term combinations were used, with database-specific field codes applied where required (Scopus: TITLE-ABS-KEY; Web of Science: TS=). Citation chaining of included studies and citing-article searches in Google Scholar were used to capture additional records. Reference lists of recent reviews [15-24] were hand-searched. An illustrative timeline of the search and screening process is provided in Figure 1.

Date Range and Geographic Restriction

Studies published between 1 January 2021 and 30 April 2026 were eligible. The window was set at five years deliberately. Almost all CBCT-specific endodontic AI work has appeared since 2021, and the underlying methods have moved quickly enough - from convolutional networks toward transformer-based segmentation - that studies published earlier no longer reflect how these systems are currently built or reported. Restricting the search to this interval keeps the synthesis aligned with the architectures now in clinical and research use and with the reporting frameworks against which the corpus was appraised (CLAIM 2024, TRIPOD-AI, QUADAS-AI). Geographic eligibility was determined from author affiliations on the published version of record, requiring the first or corresponding author to hold a primary appointment at a US or European institution. Visiting or honorary affiliations were not sufficient. This restriction was applied to align the synthesis with the FDA and European Medical Device Regulation (MDR) regulatory environments most relevant to US and European dental practitioners.

Rationale for Geographic Restriction

The review was intentionally restricted to studies with a first or corresponding author affiliated with a United States or European institution. This restriction was not intended to assess the global performance of AI in endodontics. Rather, it was designed to evaluate the evidence base most directly relevant to regulatory and clinical translation in the United States and Europe, where FDA 510(k), CE marking, and EU Medical Device Regulation pathways influence how CBCT-based dental AI tools are validated and adopted. The restriction also allowed a focused assessment of whether research groups operating within these regulatory environments have generated evidence that is sufficiently mature for clinical translation. Studies from other regions were excluded only because they fell outside this predefined scope, not because of concerns regarding scientific quality.

Selection Process

A two-stage screening was performed by the single author. Stage 1 screened titles and abstracts against modality, task, and study-design criteria. Stage 2 screened full texts against geography, AI methodology, and outcome reporting. Borderline geographic determinations were resolved by inspection of the corresponding author's email domain, ORCID, and institutional webpage.

Data Extraction

For each included study, the following were extracted: year of publication; country of corresponding author; specific endodontic task; AI architecture; dataset size in CBCT volumes and teeth; CBCT device(s) and voxel size when reported; ground-truth definition; data partitioning and validation strategy; reported performance metrics; external validation status; and code/data availability.

Software and Data Management

Records were managed and deduplicated manually in Google Sheets (Google LLC, Mountain View, USA), accessed via web browser on macOS. Screening was performed by inspecting full-text PDFs and bibliographic metadata. Descriptive calculations, including study counts, category totals, and percentage reporting-completeness scores, were performed in Google Sheets. No inferential statistical analysis or meta-analysis was performed. All figures were created by the author in Apple Keynote on macOS (Apple Inc., Cupertino, USA).

Quality Appraisal

Methodological quality was appraised against an adapted CLAIM checklist [26] and the CLAIM 2024 update [27], supplemented by the Quality Assessment of Diagnostic Accuracy Studies tailored to artificial intelligence (QUADAS-AI) principles [28,29] for studies framed as diagnostic test accuracy. Risk of bias for prediction-style models was considered against the Transparent Reporting of a Multivariable Prediction Model for Individual Prognosis or Diagnosis-Artificial Intelligence (TRIPOD-AI) [30]. Reporting completeness was evaluated using an eight-domain author-adapted framework derived from CLAIM 2024 [27] and QUADAS-AI principles [28,29]. The eight domains were: data-source description, ground-truth definition, model description, training/validation split, performance metrics, external validation, failure-mode analysis, and code/data availability. Each domain was scored as Yes, Partial, or No. For descriptive summary only, Yes was assigned 1 point, Partial 0.5 points, and No 0 points; the total was converted to a percentage. This score was not intended as a validated risk-of-bias instrument but as a structured reporting-completeness summary.

Synthesis Approach

Findings were synthesized narratively and grouped by clinical task. Performance metrics are reported as point estimates with the original precision provided by authors; ranges across studies are descriptive only and were not statistically pooled. Quality and reporting issues are discussed both within each task subsection and across the corpus.

Results

Study Selection

**Figure 2:**
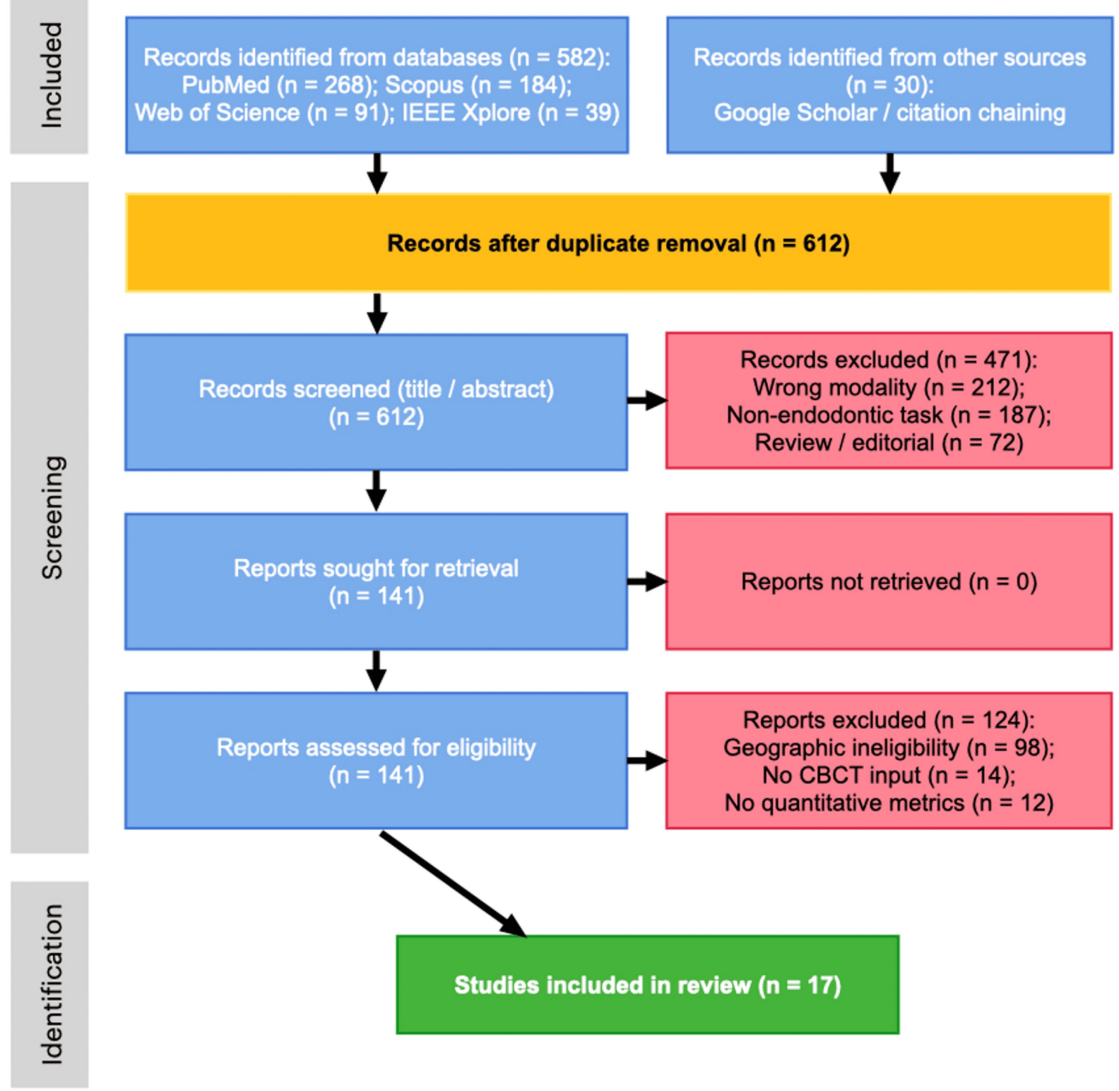
PRISMA 2020 flow diagram PRISMA 2020 flow diagram for study identification, screening, and inclusion. The figure summarizes database searching, citation chaining, duplicate removal, title and abstract screening, full-text review, and final inclusion of primary studies evaluating artificial intelligence applied to cone-beam computed tomography for endodontic tasks from January 1, 2021, through April 30, 2026. Adapted from the PRISMA 2020 flow diagram template [25]. Figure created by the author in Apple Keynote (Apple Inc., Cupertino, CA, USA) on macOS. No generative artificial intelligence was used. CBCT: cone-beam computed tomography; PRISMA: Preferred Reporting Items for Systematic Reviews and Meta-Analyses

The literature search yielded 612 unique records (PubMed 268, Scopus 184, Web of Science 91, IEEE Xplore 39, Google Scholar/citation chaining 30) after deduplication. Title/abstract screening excluded 471 records (modality, n=212; non-endodontic task, n=187; review/editorial, n=72). Full-text review excluded an additional 124 records, predominantly because of geographic ineligibility (n=98), absence of CBCT input (n=14), or lack of quantitative performance reporting (n=12). Seventeen primary studies met all inclusion criteria and form the synthesis below [6-8,31-44]. A PRISMA flow diagram is presented in Figure 2.

The corpus is dominated by four research groups: the University of Pennsylvania endodontics-engineering collaboration led by Setzer (USA, 4 studies) [31-34], the OMFS-IMPATH group at KU Leuven led by Jacobs (Belgium, 7 studies) [6-8,35-37,44], the Medical University of Graz oral surgery-computer vision collaboration led by Kirnbauer and Štern (Austria, 3 studies) [38-40], and the Stony Brook radiology-dental medicine group led by Mahdian (USA, 1 study) [41]. Two additional studies originate from a Diagnocat-Manchester collaboration [42] and from the Nicolaus Copernicus University in Poland [43]. This geographic concentration is itself a principal finding - and is discussed below (Table 1).

**Table 1:** Overview of included studies AI: artificial intelligence; BALD: Bayesian Active Learning by Disagreement; CBCT: cone-beam computed tomography; CLAHE: contrast-limited adaptive histogram equalization; CNN: convolutional neural network; DL: deep learning; FDI: Fédération Dentaire Internationale (World Dental Federation) tooth numbering system; MB2: second mesiobuccal canal; UNETR: UNEt TRansformers.

#	Study (Year), Journal	Country	Endodontic task	AI architecture
1	Lahoud et al. (2021), J Endod [6]	Belgium	Tooth segmentation	3D U-Net (Virtual Patient Creator)
2	Shaheen et al. (2021), J Dent [36]	Belgium	Multi-class tooth segmentation (FDI)	Two-stage CNN
3	Sherwood et al. (2021), J Endod [34]	USA/Germany	C-shaped canal segmentation and classification	Xception U-Net + CLAHE
4	Zheng et al. (2021), IEEE TASE [31]	USA	Periapical lesion detection	Anatomically constrained Dense U-Net
5	Ezhov et al. (2021), Sci Rep [42]	UK/international	Multi-task dental dx incl. periapical	Multi-task CNN ensemble (Diagnocat)
6	Albitar et al. (2022), Diagnostics [41]	USA	MB2 canal detection	3D U-Net
7	Fontenele et al. (2022), J Dent [35]	Belgium	Tooth segmentation under restorations	Virtual Patient Creator (validation)
8	Kirnbauer et al. (2022), J Endod [38]	Austria	Periapical lesion segmentation	Tooth localization net + 3D U-Net
9	Ver Berne et al. (2023), J Dent [44]	Belgium	Radicular cyst vs granuloma classification	DL classifier (multi-architecture)
10	Slim et al. (2024), Clin Oral Investig [7]	Belgium	Pulp cavity segmentation, mandibular molars	Virtual Patient Creator CNN
11	Hadzic et al. (2024), J Clin Med [39]	Austria	Periapical lesion non-inferiority validation	3D U-Net
12	Hadzic et al. (2024), VISAPP [40]	Austria	Tooth localization + lesion segmentation	SpatialConfiguration-Net + 3D U-Net
13	Huang et al. (2024), J Endod [32]	USA	Periapical lesion segmentation, active learning	Bayesian U-Net (BALD/Max-Entropy)
14	Chen et al. (2024), J Endod [33]	USA	Periapical lesion segmentation with transformers	Swin-UNETR vs. U-Net
15	Kazimierczak et al. (2024), J Clin Med [43]	Poland	Diagnocat commercial validation	Commercial multi-task CNN
16	Santos-Junior et al. (2025), Int Endod J [8]	Belgium	Single-rooted canal segmentation	Virtual Patient Creator CNN
17	Santos-Junior et al. (2025), Sci Rep [37]	Belgium	Maxillary premolar pulp segmentation	Virtual Patient Creator CNN

Root Canal Segmentation

**Figure 3:**
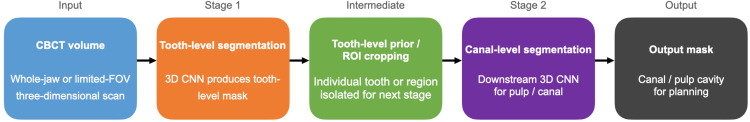
Multi-stage 3D CNN pipeline schematic Conceptual schematic of a multi-stage 3D convolutional neural network pipeline for tooth and canal segmentation on cone-beam computed tomography. The schematic illustrates how a tooth-level segmentation prior can be used to isolate an individual tooth or region of interest before downstream pulp cavity or root canal segmentation. This conceptual workflow reflects the general multi-stage approach described in the *KU Leuven studies *and is not intended to reproduce any proprietary software architecture. Figure created by the author in Apple Keynote on macOS (Apple Inc., Cupertino, USA). No generative artificial intelligence was used. CBCT: cone-beam computed tomography; CNN: convolutional neural network; FOV: field of view; ROI: region of interest.

Six studies addressed segmentation of the root canal system or pulp cavity on CBCT [6-8,35-37]. All originate from the KU Leuven group and use derivatives of a multi-stage 3D CNN pipeline deployed through the Virtual Patient Creator platform (Relu BV, Leuven, Belgium). A conceptual schematic of this multi-stage workflow is shown in Figure 3. Lahoud et al. (2021) established the foundational tooth-segmentation network on 314 CBCT volumes, achieving a mean DSC of approximately 0.97 against expert manual reference, with a more than 1,800-fold reduction in segmentation time [6]. Fontenele et al. (2022) showed that the same pipeline degrades meaningfully in the presence of metallic restorations and root canal fillings - a finding of direct endodontic relevance because endodontically treated teeth are precisely those most often re-imaged [35]. Shaheen et al. (2021) extended the framework to multi-class tooth labeling using the Fédération Dentaire Internationale (FDI, World Dental Federation) numbering system, supporting downstream automated charting [36].

Slim et al. (2024) reported pulp cavity segmentation in mandibular molars with a DSC of 0.88 ± 0.07 for first molars and 0.90 ± 0.06 for second molars, with a 95th-percentile HD of 0.13 ± 0.07 mm - significantly better than the manual operator on the HD metric (p<0.001) [7]. Santos-Junior et al. (2025, *International Endodontic Journal*) reported single-rooted tooth canal segmentation with DSC ranging from 0.89 to 0.93 and IoU from 0.80 to 0.86 across two CBCT devices, with AI inference time approximately 60-fold faster than manual segmentation [8]. A companion study in *Scientific Reports *extended the approach to maxillary premolars with comparable performance and demonstrated that second premolars were segmented more accurately than first premolars (p<0.05), reflecting reduced canal-system complexity [37].

The principal limitations are consistent: small training sets (typically 25-55 volumes), single-institution data, restricted CBCT device representation (NewTom VGi evo (Cefla, Imola, Italy) and 3D Accuitomo 170 (J. Morita, Kyoto, Japan) in nearly all KU Leuven studies), and exclusion of high-artifact scans. No study has performed prospective external validation on a fully independent cohort.

Tooth and Pulp Chamber Segmentation as Endodontic Enablers

Tooth segmentation is the necessary precursor to canal-level analysis. The Lahoud 2021 network and the Shaheen multi-class classifier [6,36] provide tooth-level priors that have been reused as preprocessing steps in subsequent canal and lesion studies. Fontenele's 2022 work [35] is methodologically important because it quantifies the failure mode most relevant to endodontists: in 95 endodontically restored teeth, mean DSC dropped to approximately 0.85, and HD increased substantially compared with intact teeth. This degradation has been only partially addressed in the published literature and remains a key barrier to deployment in real-world endodontic re-treatment planning.

A KU Leuven extension by Ver Berne et al. (2023) trained a deep-learning classifier on 215 periapical lesions to discriminate radicular cysts from periapical granulomas, achieving pilot classification accuracy that flags this as a feasible - but as yet unvalidated - adjunct to lesion detection [44].

Periapical Lesion Detection and Segmentation

Eight studies addressed periapical lesion detection or segmentation on CBCT [31-33,38-40,42-43]. Three originate from the Penn group and reflect a coherent five-year research program. Zheng et al. (2021) [31] introduced an anatomically constrained Dense U-Net trained on 20 limited-FOV CBCTs that achieved a per-root sensitivity of 0.84 and precision of 0.90, leveraging the prior that periapical lesions cluster around root apices. Huang et al. (2024, *Journal of Endodontics*) [32] implemented Bayesian U-Net with Bayesian Active Learning by Disagreement (BALD) and Max-Entropy strategies on the same 20-volume dataset, with BALD reaching 84% sensitivity and Max-Entropy reaching 96.3% specificity for the lesion class - a clinically meaningful demonstration that active learning can reduce annotation burden when expert labels are scarce. Chen et al. (2024, *Journal of Endodontics*) [33] then expanded the dataset to 138 CBCT volumes with five voxel-level labels (lesion, restorative material, bone, tooth structure, background) and compared U-Net against pretrained and from-scratch Swin-UNETR transformers; the pretrained Swin-UNETR matched or exceeded U-Net at small training-set sizes, foreshadowing transformer dominance for low-data dental tasks. False positives clustered at periodontal ligament widening, pulp chambers, and impacted teeth - an important reproducible failure mode.

The Graz group provides the most rigorous validation work. Kirnbauer et al. (2022) [38] trained a SpatialConfiguration-Net for tooth localization combined with a 3D U-Net for lesion segmentation on 144 CBCT volumes containing 206 lesions, reporting sensitivities of 92-97% and specificities of 88-88.5% across focal and focal-Tversky loss configurations. Hadzic et al. (2024, *Journal of Clinical Medicine*) [39] then performed an explicit non-inferiority validation on a single-use clinical test set of 195 CBCT scans, achieving an overall sensitivity of 86.7% and specificity of 84.3%; the null hypothesis was rejected for specificity (against an 82% margin) but not for sensitivity (against a 90% margin). The companion methods paper [40] details class-imbalance reweighting and reports stratified results by jaw, tooth type, and periapical index (PAI)-like score. This is the only US/European CBCT periapical lesion study to report a true clinical-distribution external test set with pre-specified non-inferiority margins.

A Polish single-center diagnostic accuracy study by Kazimierczak et al. (2024) [43] evaluated the commercial Diagnocat platform against three-clinician CBCT consensus on 49 patients/1,223 teeth, reporting CBCT accuracy above 94% for periapical lesion detection - substantially higher than the 33.3% sensitivity the same product showed on paired panoramic images, supporting a CBCT-specific advantage for AI-assisted apical pathosis detection. The risk of incorporation bias is non-trivial because the reference standard and one of the index modalities were both CBCT.

The Diagnocat-Manchester multi-task system reported by Ezhov et al. (2021, *Scientific Reports*) [42] was trained on 1,346 CBCTs and tested in a 24-dentist reader study on 30 CBCTs. AI assistance increased mean unaided sensitivity for periapical lesion localization from approximately 0.20 to substantially higher levels, although absolute aided sensitivity remained limited, and rare radiopaque or atypical apical anatomies were missed. This study is the most ecologically valid reader study in the corpus but does not constitute external validation in the regulatory sense.

Vertical Root Fracture Detection

No primary study with a US or European first or corresponding author was identified that applied AI to CBCT for VRF detection within the inclusion window. The principal published work in this area - Hu et al. (2022, BMC Oral Health) [45] and Yang et al. (2023, Dentomaxillofacial Radiology) [46] - originates from Chinese institutions and was excluded under the geographic filter. This represents one of the most clinically consequential gaps in the qualifying US and European literature, because VRF is the most common cause of extraction of endodontically treated teeth and is precisely the task for which CBCT is most often justified under both American Association of Endodontists/American Academy of Oral and Maxillofacial Radiology (AAE/AAOMR) [3] and ESE [4] guidance.

Root Canal Morphology Classification (C-shape, MB2, Vertucci)

Two studies directly addressed canal morphology classification. Sherwood et al. (2021), corresponding authors at Penn (Setzer) and the Charité Berlin (Schwendicke), compared U-Net, Residual U-Net, and Xception U-Net with contrast-limited adaptive histogram equalization (CLAHE) for C-shaped canal segmentation and classification in mandibular second molars on 135 limited-FOV CBCT volumes [34]. The Xception U-Net with CLAHE achieved the best segmentation overlap and classification accuracy across the three Fan or Melton C-shape configurations, although precise per-class accuracies were limited by class imbalance.

Albitar et al. (2022) at Stony Brook applied a 3D U-Net to MB2 canal detection in 102 maxillary molar roots from 57 deidentified CBCTs [41]. Detection sensitivity reached 0.80 with specificity, PPV, and NPV of 1.00, 1.00, and 0.83, respectively, although the segmentation overlap metric (mean 0.30) was modest, reflecting the small caliber of MB2 canals and the limited training set. The clinical question - whether a missed MB2 is present in an obturated maxillary molar - is binary, and the high specificity supports targeted re-treatment decision support, even at the cost of modest segmentation accuracy.

No US/European primary study was identified that performed automated Vertucci-type classification across all canal-system variants on CBCT, despite this being a foundational morphologic taxonomy.

Treatment Planning, Working Length, Outcome Prediction, Case Difficulty

This category is conspicuously empty in the US/European primary literature. No qualifying study was identified that used AI on CBCT to determine working length, predict endodontic treatment outcome, automate AAE Endodontic Case Difficulty Assessment, or detect endodontically relevant root resorption. The Herbst et al. (2023) study from Charité Berlin and LMU Munich on the prediction of root filling length used periapical radiographs rather than CBCT and was therefore excluded [47]. The Diagnocat-affiliated multi-task system [42] touches treatment planning indirectly through segmentation outputs but does not produce explicit outcome predictions.

Cross-Cutting Findings

**Figure 4:**
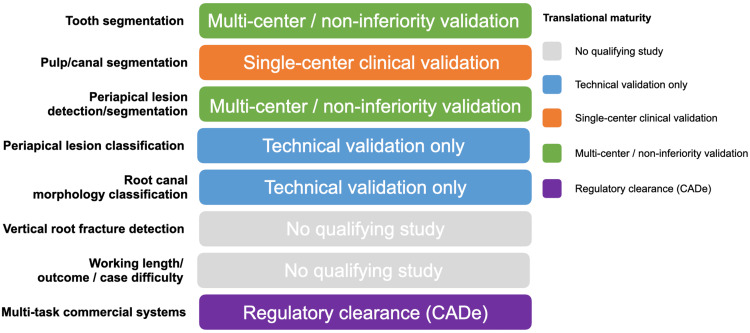
Task taxonomy and translational maturity heat map Task taxonomy and translational maturity of artificial intelligence applications to cone-beam computed tomography in endodontics. The heat map summarizes eight endodontic task categories and the highest level of translational maturity reached by any qualifying US or European primary study within the inclusion window. Task categories are consolidated relative to Table 2 to provide a higher-level overview of translational maturity; the included-study counts shown in Table 2 sum to the totals reflected here. Light gray indicates no qualifying study identified. Blue indicates technical validation only. Orange indicates single-center clinical validation. Green indicates multicenter or non-inferiority validation. Purple indicates regulatory clearance with a relevant labeled indication. Figure created by the author in Apple Keynote on macOS (Apple Inc., Cupertino, USA). No generative artificial intelligence was used. CADe: computer-aided detection

Several patterns emerge across the corpus. Performance ranges by task category are summarized in Table 2. Architectures are dominated by U-Net and 3D U-Net derivatives (13/17 studies); transformer-based architectures (Swin-UNETR) appear only in the Penn 2024 work [33] and remain a minority approach. Dataset sizes are small by general medical-AI standards: median training-set size is approximately 30 CBCT volumes (interquartile range 20-55), with the largest training dataset being 1,346 volumes for the Diagnocat multi-task system [42]. External validation is performed in only a single study (Hadzic et al. 2024, *J Clin Med*) [39]. Code and data availability are poor: fewer than one-third of studies provide a public repository, and no study releases full annotated CBCT volumes, principally because of patient privacy constraints. CBCT device diversity is limited; most studies use one or two scanners, and voxel size, FOV, and exposure parameters are inconsistently reported. Reporting completeness against CLAIM 2024 averages approximately 60-70% of applicable items (Table 3), with the most consistent gaps in sample-size justification, ground-truth uncertainty quantification, and failure-mode analysis. Dataset characteristics for each study, including the CBCT devices used, are detailed in Table 4. The translational maturity reached by each task category is summarized in Figure 4.

**Table 2:** Performance ranges by task category Performance ranges across the included studies by task category, including the number of contributing studies and the reported Dice similarity coefficient, sensitivity, and specificity ranges. Ranges are descriptive across the included primary studies [6-8,31-44] and were not statistically pooled because of clinical and methodological heterogeneity. The eight task categories sum to the 17 included studies; the "Multi-task commercial systems" category overlaps clinically with periapical lesion detection and segmentation because the included commercial systems output periapical findings as one of their primary tasks. DSC: Dice similarity coefficient; MB2: second mesiobuccal canal; n/r: not reported.

Task category	Studies (n)	DSC range	Sensitivity range	Specificity range
Tooth segmentation	3	0.85 - 0.97	n/r	n/r
Pulp / canal segmentation, single-rooted	1	0.89 - 0.93	n/r	n/r
Pulp / canal segmentation, multi-rooted	2	0.88 - 0.90	n/r	n/r
Periapical lesion detection / segmentation	6	n/r	0.80 - 0.97	0.84 - 1.00
Periapical lesion classification (cyst vs. granuloma)	1	n/r	n/r	n/r
C-shaped canal classification	1	DSC reported	n/r	n/r
MB2 canal detection	1	0.30 (mean)	0.80	1.00
Multi-task commercial systems	2	n/r	Per-task; aided gain shown	n/r

**Table 3:** Reporting and methodological quality appraisal of included studies, mapped against CLAIM 2024 and QUADAS-AI Reporting and methodological quality appraisal of the included primary studies [6-8,31-44], mapped against eight domains adapted from CLAIM 2024 and QUADAS-AI principles [26-29]. Yes = item fully met or reported; Partial = item partially met (for example, one annotator only, or partial split reported); No = item not reported or not met. Score (%) = (Yes + 0.5 x Partial) / 8 items, rounded to the nearest whole percent. This scoring framework is a structured reporting-completeness summary and is not a validated risk-of-bias instrument. CLAIM: Checklist for Artificial Intelligence in Medical Imaging; QUADAS-AI: Quality Assessment of Diagnostic Accuracy Studies tailored to artificial intelligence.

#	Study	Data source described	Ground truth defined	Model described	Train/val split	Performance metrics	External validation	Failure analysis	Code/data available	Score (%)
1	Lahoud 2021 [6]	Yes	Yes	Yes	Yes	Yes	No	Partial	No	69%
2	Shaheen 2021 [36]	Yes	Yes	Yes	Yes	Yes	No	No	No	63%
3	Sherwood 2021 [34]	Yes	Yes	Yes	Yes	Yes	No	Partial	No	69%
4	Zheng 2021 [31]	Yes	Yes	Yes	Yes	Yes	No	Yes	No	75%
5	Ezhov 2021 [42]	Yes	Yes	Yes	Yes	Yes	Partial	Yes	No	81%
6	Albitar 2022 [41]	Yes	Yes	Yes	Yes	Yes	No	Partial	No	69%
7	Fontenele 2022 [35]	Yes	Yes	Yes	Yes	Yes	No	Yes	No	75%
8	Kirnbauer 2022 [38]	Yes	Yes	Yes	Yes	Yes	No	Yes	No	75%
9	Ver Berne 2023 [44]	Yes	Yes	Yes	Yes	Yes	No	Partial	No	69%
10	Slim 2024 [7]	Yes	Yes	Yes	Yes	Yes	No	Partial	No	69%
11	Hadzic 2024 (JCM) [39]	Yes	Yes	Yes	Yes	Yes	Yes	Yes	No	88%
12	Hadzic 2024 (VISAPP) [40]	Yes	Yes	Yes	Yes	Yes	Partial	Yes	No	81%
13	Huang 2024 [32]	Yes	Yes	Yes	Yes	Yes	No	Yes	No	75%
14	Chen 2024 [33]	Yes	Yes	Yes	Yes	Yes	No	Yes	No	75%
15	Kazimierczak 2024 [43]	Yes	Yes	Yes	Yes	Yes	No	Partial	No	69%
16	Santos-Junior 2025 (IEJ) [8]	Yes	Yes	Yes	Yes	Yes	No	Partial	No	69%
17	Santos-Junior 2025 (SR) [37]	Yes	Yes	Yes	Yes	Yes	No	Partial	No	69%

**Table 4:** Dataset characteristics across included studies Dataset characteristics across the 17 included primary studies [6-8,31-44], including number of CBCT volumes, number of teeth or lesions, and CBCT device(s) used. Data were extracted directly from the Methods sections of the original publications; entries marked with an em dash indicate that the original publication did not specify the parameter. CBCT: cone-beam computed tomography; FOV: field of view; IEJ: *International Endodontic Journal*; JCM: *Journal of Clinical Medicine*; SR: *Scientific Reports*; VISAPP: International Conference on Computer Vision Theory and Applications.

#	Study	CBCT volumes	Teeth/lesions	CBCT device(s)
1	Lahoud 2021 [6]	314 vols	—	NewTom VGi evo (Cefla, Imola, Italy)
2	Shaheen 2021 [36]	433 vols	—	NewTom VGi evo (Cefla, Imola, Italy)
3	Sherwood 2021 [34]	135 vols	135 teeth	Limited-FOV (vendor not reported)
4	Zheng 2021 [31]	20 vols	20+ teeth	Carestream limited-FOV (Carestream Dental, Atlanta, GA, USA)
5	Ezhov 2021 [42]	1,346 train / 30 test	—	Multiple commercial scanners
6	Albitar 2022 [41]	57 vols	102 roots	Carestream CS 9300 (Carestream Dental, Atlanta, GA, USA)
7	Fontenele 2022 [35]	100 vols	146 teeth	NewTom VGi evo (Cefla, Imola, Italy)
8	Kirnbauer 2022 [38]	144 vols	206 lesions	ProMax 3D Mid (Planmeca, Helsinki, Finland)
9	Ver Berne 2023 [44]	—	215 lesions	Multiple
10	Slim 2024 [7]	116 vols	116 mand. molars	NewTom VGi evo (Cefla, Imola, Italy)
11	Hadzic 2024 (JCM) [39]	195 vols	—	ProMax 3D Mid (Planmeca, Helsinki, Finland)
12	Hadzic 2024 (VISAPP) [40]	144 vols	206 lesions	ProMax 3D Mid (Planmeca, Helsinki, Finland)
13	Huang 2024 [32]	20 vols	20+ teeth	Carestream (Carestream Dental, Atlanta, GA, USA)
14	Chen 2024 [33]	138 vols	—	Carestream limited-FOV (Carestream Dental, Atlanta, GA, USA)
15	Kazimierczak 2024 [43]	49 vols	1,223 teeth	Single vendor (reported)
16	Santos-Junior 2025 (IEJ) [8]	—	Per-tooth	NewTom VGi evo (Cefla, Imola, Italy) + 3D Accuitomo 170 (J. Morita, Kyoto, Japan)
17	Santos-Junior 2025 (SR) [37]	—	Maxillary premolars	NewTom VGi evo (Cefla, Imola, Italy)

Discussion

Principal Findings

The central contribution of this review is a task-level map of CBCT endodontic AI in the United States and Europe showing which tasks have reached clinical maturity and which remain unaddressed (Figure 4). The US and European primary evidence base for AI applied to CBCT in endodontics over the 2021-2026 window comprises a small but methodologically coherent body of work, dominated by four research groups and concentrated on three tasks: tooth and pulp segmentation, periapical lesion detection, and selected morphologic classification problems. Pulp and root canal segmentation in single- and multi-rooted teeth has reached high technical performance on benchmark datasets, with DSCs above 0.88 across multiple anatomies and validated reductions in operator time of one to two orders of magnitude. Periapical lesion detection has reached early clinical validation, with one study (Hadzic et al. 2024) [39] meeting a pre-specified non-inferiority specificity threshold on an independent clinical test set. Other endodontically critical tasks - VRF detection, working length determination, AAE difficulty assessment, treatment outcome prediction - are essentially absent from the qualifying US and European literature.

State of Clinical Translation

The regulatory landscape for CBCT-based dental AI has evolved during the inclusion window. Between 2024 and early 2026, the US Food and Drug Administration (FDA) cleared several 510(k) products from Pearl, Overjet, and Diagnocat. These clearances cover CBCT-based anatomical visualization, segmentation, and measurement. At least one product has additionally received clearance to function as a concurrent-read computer-aided detection (CADe) aid for periapical radiolucency on CBCT in permanent teeth [14]. The labeled indications across these products consistently emphasize that the software is intended to assist clinicians' review and is not a substitute for professional interpretation or autonomous diagnostic decision-making. Authors and users are encouraged to consult the FDA 510(k) database for the current scope of each device's labeled indication, as the field is changing rapidly.

Even with these clearances, the published primary studies that come closest to clinical readiness have not been translated into commercially available products with US labeling that matches their academic scope. This is true at the time of writing for the Penn periapical lesion segmentation series [31-33] and for the Graz validation work [38-40]. The Virtual Patient Creator platform (Relu BV) underlying the KU Leuven work is CE-marked under the EU MDR for tooth and pulp segmentation in treatment planning workflows but is not approved for autonomous diagnostic decision-making.

This gap between research performance and regulatory clearance is consistent with the general pattern of medical AI translation. It is exaggerated in dentistry, where regulatory pathways for diagnostic AI are still maturing and where prospective multi-center evidence is rarely generated outside of industry-sponsored programs.

The distinction between technical validation and clinical validation is particularly important for CBCT-based dental AI. High Dice scores or sensitivity values obtained on curated single-center datasets do not necessarily imply safe clinical deployment, because CBCT image quality varies by scanner, field of view, voxel size, exposure protocol, patient motion, and metallic artifact burden [27-30,35]. Future studies should therefore report not only aggregate model performance but also scanner-level and artifact-level subgroup analyses, failure modes, and prospective reader-impact outcomes.

Methodological Strengths and Weaknesses

Strengths include the consistent use of expert manual segmentation as the reference standard, increasing adoption of 3D rather than 2D-slice approaches, and growing methodological sophistication (active learning [32], transformer pretraining [33], anatomically constrained losses [31], class-imbalance reweighting [40]). The Graz group's pre-specified non-inferiority validation [39] is exemplary and should be widely emulated.

Weaknesses are substantial. Dataset sizes remain small, as quantified above; reporting against CLAIM 2024 [27] is incomplete, particularly for sample-size justification, robustness analyses, and failure-mode characterization; external validation across CBCT devices, voxel sizes, and FOVs is rare; and no qualifying study performs prospective patient-level validation in a clinical workflow. These generalizability concerns are not theoretical: Fontenele et al. showed that metallic restorations and partial-volume effects measurably degrade CBCT segmentation performance [35].

Reporting Quality

When mapped against CLAIM 2024 [27] and TRIPOD-AI [30], the corpus shows three recurrent reporting gaps. First, ground-truth uncertainty is rarely quantified - interobserver agreement among annotators is reported in fewer than half of the studies, and intra-observer agreement almost never. Second, dataset provenance is opaque: scanner-level, FOV-level, and operator-level distributions of the training and test data are seldom disclosed, making reproducibility and external generalization assessment difficult. Third, code and trained-model availability is the exception rather than the rule - in part because of legitimate privacy constraints on CBCT data, but also because of journal and institutional incentive structures that do not yet require open release.

Comparison With Systemic Medicine AI Maturity

In contrast to ophthalmology (diabetic retinopathy screening) or radiology (pulmonary nodule detection, intracranial hemorrhage triage), where multi-center prospective trials and FDA clearances with autonomous diagnostic indications now exist, dental AI on CBCT remains predominantly at the technical-validation rather than clinical-validation stage. As noted, the only external validation in this corpus [39] and the single multi-reader study [42] are encouraging but isolated. Closing that gap remains an open challenge: it will require coordinated multi-center datasets, harmonized acquisition protocols, and prospective clinical-utility endpoints - change in management, treatment outcome, time saved - rather than purely metric-based endpoints.

Limitations of This Review

This review has several limitations. First, the strict US/European geographic filter excludes a large body of high-quality work from Chinese, Korean, Japanese, Turkish, and Brazilian groups, including the Wuhan PAL-Net 3D CNN and the leading Asian VRF and root resorption studies. The synthesis is therefore not a global account of the field; it is a deliberate audit of US and European evidence in a regulatory context. Second, narrative synthesis without quantitative pooling does not produce summary effect estimates with confidence intervals; given the heterogeneity observed, however, pooling would not have been methodologically appropriate. Third, English-language restriction may have missed European-language conference proceedings, although the major venues - the Medical Image Computing and Computer-Assisted Intervention conference (MICCAI), the IEEE Engineering in Medicine and Biology Conference (EMBC), SPIE Medical Imaging, and the International Conference on Computer Vision Theory and Applications (VISAPP) - publish in English. Fourth, the corpus is small enough that publication bias toward positive results, while not formally testable, is plausible. Finally, this review was conducted by a single author; while two-stage screening was systematically applied, dual independent screening would have provided stronger procedural rigor.

Future Research Priorities

Seven priorities emerge from this synthesis. First, multi-center, multi-device CBCT datasets with harmonized annotation protocols are urgently needed; the current single-institution paradigm cannot support clinical translation. Second, vertical root fracture detection on CBCT in US and European cohorts is a critical unaddressed task and should be a near-term focus, ideally as a prospective study with surgical or extraction-based reference standards. Third, treatment outcome prediction that integrates pre-treatment CBCT features with clinical and microbiological covariates would offer high clinical value and is methodologically tractable with TRIPOD-AI-aligned modeling. Fourth, AAE Endodontic Case Difficulty Assessment automation is a low-risk, high-utility task that could be pursued with existing tooth and canal segmentation infrastructure. Fifth, transformer architectures and self-supervised pretraining on unlabeled CBCT volumes deserve systematic investigation given the small annotated datasets that characterize this field. Sixth, prospective reader studies with pre-specified clinical-utility endpoints - change in diagnosis, change in treatment plan, time to diagnosis - should replace metric-only validation in mature tasks. Seventh, the field should adopt CLAIM 2024 and TRIPOD-AI as minimum reporting standards, with journals and institutional review boards encouraged to require deposition of trained models and de-identified test data wherever privacy permits.

## Conclusions

Cone-beam computed tomography-based AI in endodontics has matured unevenly across tasks in US and European centers between 2021 and 2026. Tooth and pulp cavity segmentation shows high technical performance on benchmark datasets, with DSCs above 0.88 across single- and multi-rooted teeth and substantial time savings versus manual annotation. Periapical lesion detection has progressed to the point of single-site non-inferiority validation, with sensitivities and specificities in the mid-80% range on independent clinical data. Morphologic classification of C-shaped canals and detection of MB2 anatomy are early but credible. Vertical root fracture detection, working length determination, case difficulty assessment, and treatment outcome prediction on CBCT remain essentially unaddressed in the qualifying US and European literature, despite each being a high-value clinical question explicitly named in AAE/AAOMR and ESE guidance. Clinicians should approach current FDA-cleared CBCT products with awareness of each device's specific labeled indication and should not yet expect autonomous endodontic interpretation. Researchers should prioritize multi-center datasets, prospective clinical-utility endpoints, and adherence to CLAIM 2024 and TRIPOD-AI reporting standards. Bridging that gap - between high technical performance on small single-center datasets and prospective clinical validation in heterogeneous environments - remains the central translational task of the next five years.
